# A potential role for intragenic miRNAs on their hosts' interactome

**DOI:** 10.1186/1471-2164-11-533

**Published:** 2010-10-01

**Authors:** Ludwig Christian G Hinske, Pedro AF Galante, Winston P Kuo, Lucila Ohno-Machado

**Affiliations:** 1 Department of Anaesthesiology, Clinic of the University of Munich, Marchioninistrasse 15, 81377 Munich, Germany; 2 Division of Biomedical Informatics, University of California San Diego, 9500 Gilman Dr, La Jolla, California 92093, USA; 3 Ludwig Institute for Cancer Research, Hospital Alemão Oswaldo Cruz, Rua João Julião, São Paulo 01323-903, Brazil; 4 Harvard School of Dental Medicine, 188 Longwood Avenue, Boston, Massachusetts 02115, USA; 5 Harvard Catalyst - Laboratory for Innovative Translational Technologies, Harvard Medical School, 77 Avenue Louis Pasteur, Boston, Massachusetts 02115, USA

## Abstract

**Background:**

miRNAs are small, non-coding RNA molecules that mainly act as negative regulators of target gene messages. Due to their regulatory functions, they have lately been implicated in several diseases, including malignancies. Roughly half of known miRNA genes are located within previously annotated protein-coding regions ("intragenic miRNAs"). Although a role of intragenic miRNAs as negative feedback regulators has been speculated, to the best of our knowledge there have been no conclusive large-scale studies investigating the relationship between intragenic miRNAs and host genes and their pathways.

**Results:**

miRNA-containing host genes were three times longer, contained more introns and had longer 5' introns compared to a randomly sampled gene cohort. These results are consistent with the observation that more than 60% of intronic miRNAs are found within the first five 5' introns. Host gene 3'-untranslated regions (3'-UTRs) were 40% longer and contained significantly more adenylate/uridylate-rich elements (AREs) compared to a randomly sampled gene cohort. Coincidentally, recent literature suggests that several components of the miRNA biogenesis pathway are required for the rapid decay of mRNAs containing AREs. A high-confidence set of predicted mRNA targets of intragenic miRNAs also shared many of these features with the host genes. Approximately 20% of intragenic miRNAs were predicted to target their host mRNA transcript. Further, KEGG pathway analysis demonstrated that 22 of the 74 pathways in which host genes were associated showed significant overrepresentation of proteins encoded by the mRNA targets of associated intragenic miRNAs.

**Conclusions:**

Our findings suggest that both host genes and intragenic miRNA targets may potentially be subject to multiple layers of regulation. Tight regulatory control of these genes is likely critical for cellular homeostasis and absence of disease. To this end, we examined the potential for negative feedback loops between intragenic miRNAs, host genes, and miRNA target genes. We describe, how higher-order miRNA feedback on hosts' interactomes may at least in part explain correlation patterns observed between expression of host genes and intragenic miRNA targets in healthy and tumor tissue.

## Background

microRNAs (miRNAs) are small (~22-nt) functional RNA species that provide a newly appreciated layer of gene regulation with an important role in development, cellular homeostasis and pathophysiology. miRNAs are encoded in the genome and transcribed primarily in a Pol II-dependent manner [1], although Pol III-dependent transcription has also been reported [2,3]. Roughly half of the known human microRNAs are found in intergenic regions of the genome, suggesting production of unique primary transcripts (pri-miRNAs) containing one or more miRNA hairpins under the control of independent promoter elements. The overwhelming majority of the other ~50% map to previously annotated intronic regions of protein coding genes, while a small number are even found within exons. The relationship between intragenic miRNAs and their host genes presents many unique questions regarding genomic organization, transcriptional regulation, processing and function.

The genomic organization of intragenic miRNAs exhibits a strong directional bias, such that these species are predominantly oriented on the same strand of the DNA as that of the host gene. The directional bias may prevent steric interference between RNA polymerases transcribing the host gene and the miRNA gene(s) [4]; however, the existence of individual antisense miRNA genes and miRNA gene clusters argues that the primary evolutionary pressure for the positional bias is co-regulation of the intronic miRNA and the host gene. Indeed, microarray analyses supports the hypothesis that intronic miRNAs are usually expressed in coordination with the host gene mRNA in human tissues [4,5], strongly suggesting that co-transcription from the host gene promoter is the most common transcriptional mechanism under normal conditions. This assumption has lately successfully been employed to identify new miRNA targets [6]. However, recent findings demonstrate that transcription of a subset of intronic miRNAs in *H. sapiens *can be initiated from internal promoters within operons independently from the host gene [3], suggesting that utilization of internal promoters must also be considered a viable alternative strategy for intronic miRNA gene transcription.

Large portions of miRNA processing are understood (for review see [7]). In brief, a ~70 nucleotide stem-loop precursor pre-miRNA is excised from a relatively long primary miRNA transcript, followed by export from the nucleus via Exportin-5 in a Ran-GTP-dependent manner. In the cytoplasm, pre-miRNAs are further processed into a ~22-nt miRNA/miRNA* duplex. In the case of intronic miRNAs, early steps in the miRNA biogenesis pathway are complicated by the requirement for proper pre-mRNA splicing and mature mRNA assembly of the host message. Recent bioinformatics and experimental work demonstrates that intronic miRNAs can be processed from intronic regions co-transcriptionally [8] prior to the splicing reaction [9]. Interestingly, recent work suggests that several intragenic miRNAs undergo post-transcriptional regulation [10], and defects in this process have been associated with tumor development [10-14]. The nature of the differences in miRNA processing and associated defects between intergenic and intragenic miRNA species is not currently elucidated.

miRNA target recognition in mammals is mainly mediated via imperfect Watson-Crick base-pairing to cognate sites primarily located in the 3'-UTR of mRNA targets. Predicted and validated miRNA targets include a functionally diverse suite of genes that include many transcription factors and cell signaling proteins, suggesting a role for miRNAs in regulatory feedback loops [15-17]. Intragenic miRNAs present unique regulatory possibilities based on functional relationships with their host genes. It has been speculated that intronic miRNAs may directly target their host message or regulate transcription factors, in what is commonly designated "first-order" or "second-order" negative feedback, respectively [18]. Recently published work [19] demonstrates that miR-338, encoded in an intron of the apoptosis-associated tyrosine kinase (AATK) gene, targets several genes that are functionally antagonistic to the AATK protein. Therefore, miR-338 serves the functional interest of the host in this case via a higher-order positive feedback system that downregulates expression of AATK repressors and enforces neuronal differentiation downstream of the kinase.

In the current manuscript, large-scale bioinformatics analyses of human intronic miRNAs related to genomic organization and characterization of miRNA host and target genes are presented. We identify characteristics of host genes and predicted targets, and present evidence that intragenic miRNAs may act as negative feedback regulatory elements of their hosts' interactome (i.e., they can regulate host gene neighbours in addition to host genes).

## Results

We integrated genomic and transcriptomic information to analyze properties of intragenic miRNAs themselves, their host genes, as well as their targets. We used all known miRNAs (based on miRBase), all known human transcripts (based on RefSeq), six different and highly established miRNA target prediction algorithms, as well as the gene and pathway annotation ontologies GO and KEGG.

### Classification of miRNAs

Based on mapping miRNA genomic coordinates to genomic position of all known genes and their exons and introns (based on RefSeq sequences [20]), we could classify miRNAs into three classes: intergenic, exonic, and intronic (Table 1). For *H. sapiens*, 296 miRNAs were located within intronic regions, and 37 within exonic regions of known genes. We also classified miRNAs from other species (Table 1). Interestingly, organisms that have a well-annotated set of protein-coding genes present distributions that resemble that of the miRNA distribution in humans, showing 33-48% of intronic miRNAs and 0.6-6% of exonic miRNAs (Table 1, organisms *M. musculus, D. melanogaster *and *C. elegans*). On the other hand, organisms containing a smaller number of annotated genes presented a higher number of intergenic miRNAs (Table 1, organisms *C. familiaris, G. gallus *and *D. rerio*), some of which however may become intragenic as more genes will be identified in these organisms. Additional file 1 contains details of miRNAs classification, their genomic position and host genes.

**Table 1:** Classification of miRNAs in the Genome of Different Species

Organism	Intragenic miRNAs		Intergenic miRNAs	Intragenic miRNAs	
			
	Intronic	Exonic		miRNAs on Host Gene Strand	miRNAs on Opposite Host Strand
*Homo sapiens*	296 (42.6%)	37 (5.3%)	362 (52.1%)	282 (84.7%)	51 (15.3%)

*Mus musculus*	171 (35.4%)	30 (6.2%)	282 (58.4%)	163 (78.2%)	38 (21.8%)

*Canis familiaris*	3 (1.5%)	0 (0%)	201 (98.5%)	2 (66.7%)	1 (33.3%)

*Gallus gallus*	50 (10.7%)	1 (0.2%)	418 (89.1%)	46 (90.2%)	5 (9.8%)

*Danio rerio*	48 (15.0%)	1 (0.3%)	271 (84.7%)	39 (79.6%)	10 (20.4%)

*Drosophila melanogaster*	65 (42.8%)	2 (1.3%)	85 (55.9%)	53 (79.1%)	14 (20.9%)

*Caenorhabditis elegans*	51 (33.1%)	1 (0.6%)	102 (66.2%)	33 (63.6%)	19 (36.5%)

Intragenic miRNAs are found in many different species. However, the distribution of intra- and intergenic miRNAs differs. These numbers are obtained by crossing miRNA genomic coordinates with known transcript coordinates (based on RefSeq sequences).

### Positional Bias of Intragenic miRNAs

The orientation of the gene for an intronic miRNA depends significantly on the transcription direction of its host gene (p-value = 1.3 × 10-36 in χ^2 ^test) as shown in Table 1. We found that 65.5% of host genes had miRNAs in the first five introns. Also, we confirmed that the observed distribution differs significantly from the expected distribution within the first five introns (p = 0.030 in χ^2 ^test, additional file 2).

### Characterization of Host Genes

**Figure 1:**
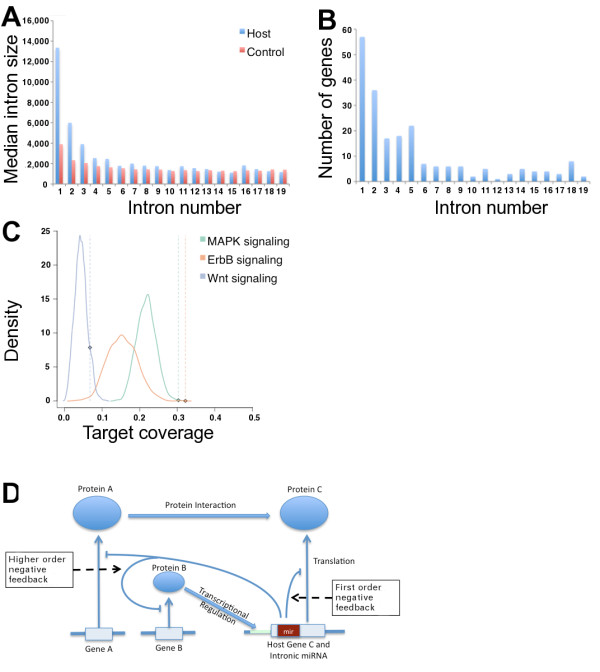
**Intragenic miRNA Properties**. (a) The sizes of host gene introns closest to the TSS are significantly larger than those of respective controls. (b) Intronic miRNAs appear to be unevenly distributed across the intronic regions of their host genes. More then half of intronic miRNAs are located within the first five 5' introns of their hosts. (c) The observed target coverage (diamond) is at the upper end of the random distribution of target coverages for different signaling pathways, indicating that intragenic miRNAs have more targets within the pathway than would be expected by chance. (d) Intragenic miRNAs may control their host in the setting of a negative feedback circuitry not only through direct regulation of the host's transcription, but also on the order of the interactome, by controlling other genes in the host's pathway.

Assuming, as is widely accepted, that intragenic miRNAs share a common regulatory control with their host genes, we can infer functional aspects of this class of miRNAs by characterizing features of those host genes. To confirm that the position of miRNAs has a particular bias, and is not the result of chance, we randomly sampled genes that matched the set of miRNA host genes (in terms of chromosome and strand distribution) and compared the positions of host, target, and randomly sampled genes. The findings are summarized in Table 2. Host genes are almost three times longer than the randomly sampled cohort and have more introns. When comparing the intron size in different positions (Figure 1a), we found that the first five 5' introns are significantly longer, consistent with our previous finding that most host genes' intronic miRNAs are found in the 5' introns (Figure 1b).

**Table 2:** Properties of Host and Target Genes

Property	Gene Set	Median[Range] Host/Target	Median[Range] Control	Ratio	p-Value
Total length (basepairs)	Host Genes	84871.0 [2792-2220381]	29324.5 [599-2304633]	2.89	< 2.2e-16
	
	Target genes	83747.5 [2366-2220381]	30232.5 [218-2220381]	2.77	< 2.2e-16

Introns	Host Genes	13[1-88]	8[1-105]	1.62	4.3e-13
	
	Target genes	10.5[0-78]	8[0-311]	1.31	9.77e-07

Length 5'UTR (basepairs)	Host Genes	279.5[0-385608]	298.5[0-1098107]	0.94	0.25
	
	Target genes	439.5[0-460277]	282.5[0-1098107]	1.56	2.32e-08

Length 3'UTR (basepairs)	Host Genes	1218.5[0-535884]	872[0-321862]	1.4	4.71e-05
	
	Target genes	1764[171-11799]	872[0-72058]	2.2	< 2.2e-16

ARE (absolute)	Host Genes	2.0[0-1794]	1.0[0-592]	2.0	3.89e-04
	
	Target genes	5.0[0-47]	2.0[0-187]	2.5	< 2.2e-16

ARE (per kb)	Host Genes	1.9[0-2.74]	1.49[0-0.045]	1.26	0.012
	
	Target genes	2.69[0-14.22]	1.63[0-76.92]	1.65	< 2.2e-16

5' UTR GC content	Host Genes	0.6[0.31-0.95]	0.59[0-1]	1.06	0.015
	
	Target genes	0.59[0.26-1]	0.58[0-1]	1.01	0.71

Host and target genes display similar properties, compared to a set of control genes, including increased length, higher number of total introns, longer 3'UTRs and higher frequency of "AU-rich elements" (AREs).

Gene expression can be pre- and post-transcriptionally controlled through regulatory motifs in their 3'-UTRs. Even though regulatory mechanisms are not well understood, two important concepts include regulation through miRNAs, and the role of adenylate/uridylate-rich elements (AREs) in mediating mRNA decay, which plays a significant role in cancer development [21-23]. We first compared the length of the 3'-UTRs of host genes to the length of 3'-UTRs of the random sample. Host genes have 40% longer 3'-UTRs (*p-value *< 0.01). In a second step, we counted occurrences of the pentamer AUUUA in these regions, normalized by the length of the 3'-UTRs. We found significantly more ARE units in host genes than in the random sample (*p-value *< 0.01). Since recently miRNA target genes have been shown to be larger than non-target genes [24], we analyzed total lengths and lengths of 3'-UTRs [25] for host genes predicted to be targeted by their intragenic miRNA and the remaining host genes separately. No significant difference in lengths between the two groups of host genes was observed (*p-value *= 0.3939), but genes in both groups were longer than genes in the control group (*p-value *= 1.552e-07 and *p-value *< 2.2e-16).3'-UTRs were longer in host genes predicted to be targets of their intronic miRNA than in host genes not predicted to be targets (*p-value *= 0.001) and control genes (*p-value *= 5.012e-07). In contrast, the 5'-UTRs of host genes were not significantly longer than the ones in the control group (*p-value *> 0.05).

The GO Biological Process (GOBP) and KEGG are ontologies that associate genes with, cellular processes and biochemical pathways, respectively, including disease pathways. When surveying GOBP for overrepresentation of miRNA host genes in certain categories, we found significant enrichment in gene regulatory, metabolic, neurogenic, and cytoskeletal processes, which reflects the broad range of diseases with which miRNAs have been associated [12,26-34]. Additionally, we found that host genes were overrepresented in several signaling pathways, such as the *MAPK*, *ErbB*, *VEGF*, and the calcium signaling pathway.

### Genomic Properties of Target Genes

We looked at genomic properties of a high-confidence set of targets for hosts of intronic miRNAs (prediction agreement ≥ 6) that would give us a set of similar size as the host genes. We then randomly sampled RefSeq transcripts to match chromosome and strand distribution as a control set and performed the analysis analogously to the analysis of genomic properties of the host genes themselves. Table 2 summarizes the results, revealing that the predicted targets have properties that are highly similar to those of host genes.

### Relationship Between Intragenic miRNAs and Host Genes

We found that approximatelly 20% of intragenic miRNAs (56 of them, hosted in 49 distinct genes) are predicted to target their own host by at least two methods. This number is significantly higher than would be expected by chance alone (*p-value *< 0.001, obtained by random sampling). Furthermore, we assessed the robustness of our approach by following the above procedure while applying a voting method as the gold standard. We assigned each of the target prediction methods to one of two groups of equal size (n = 3) and required at least one vote from each group to consider that a prediction of a miRNA-host interaction. TarBase did not contain a single instance of miRNA-host interaction, so it was excluded from the analysis. Although the numbers of miRNAs predicted to target their own host varied (12 - 55), depending on which group they had been assigned to, in each case the observed number was significantly higher than would be expected by chance (*p-value *< 0.05, see also additional file 3). Given that host genes that were predicted to be targets of their intragenic miRNA have longer 3'-UTR regions, statistical significance of the number of hosts being targeted by their intronic miRNAs was assessed by repeated creation of sets of non-host control genes with similar 3'-UTR distribution (see Materials and Methods). In line with our previous observations, the number of hosts predicted to be targeted by their intragenic miRNAs (49) was significantly higher than expected by chance (*p-value *= 0.032).

In order to test the hypothesis that intronic miRNAs might act as regulators even in the global functional context of a negative feedback loop circuitry, the KEGG pathway analysis was extended to identify targets within the respective biomolecular pathway. We defined the target coverage as the number of genes within a pathway that were predicted targets (prediction agreement ≥ 2) of miRNAs residing in host genes within that pathway, over the total number of genes in the pathway. To check whether the observed target coverage could be expected by chance, the original genes contained in the pathway were replaced by a set of randomly sampled genes and the expected target coverage of intronic miRNAs with host genes in a particular pathway was calculated. The distributions of expected target coverage for three signaling pathways are visualized in Figure 1c. At a false discovery rate (FDR) of 10%, 22 out of 74 pathways with which host genes were associated showed a significant overrepresentation of targets in the hosts' pathways (Table 3, Additional File 5). Interestingly, many signalling and malignancy-related pathways ranked high.

**Table 3:** Pathways with Overrepresentation of Genes Targeted by an Intronic miRNA

Pathway	Host Genes in Pathway	Target Coverage	p-Value	q-Value
MAPK Signaling	ATF2; DDIT3; AKT2; FGF13; ARRB1; PPP3CA; PRKCA; CACNG8; RPS6KA2; MAP2K4; RPS6KA4	61.4%	< 0.001	< 0.001

Axon Guidance	PPP3CA; PTK2; SEMA4G; SEMA3F; SLIT3; ABLIM2; SLIT2	70.3%	< 0.001	< 0.001

Ubiquitin Mediated Proteolysis	HUWE1; WWP2; BIRC6; ITCH	53.8%	< 0.001	< 0.001

Focal Adhesion	COL3A1; AKT2; PRKCA; PTK2; TLN2	49.5%	< 0.001	< 0.001

Glioma	AKT2; PRKCA	52.3%	< 0.001	< 0.001

Melanoma	AKT2; FGF13	50.7%	< 0.001	< 0.001

Regulation of Actin Cytoskeleton	CHRM2; FGF13; SSH1; PTK2	41.0%	< 0.001	< 0.001

Chronic Myloid Leukemia	AKT2	38.2%	< 0.001	< 0.001

Colorectal Cancer	AKT2	35.7%	< 0.001	< 0.001

Prostate Cancer	AKT2	34.8%	0.001	0.007

Melanogenesis	PRKCA	21.6%	0.001	0.007

Pancreatic Cancer	AKT2	35.6%	0.002	0.01

ErbB Signaling	ERBB4; AKT2; PRKCA; PTK2; MAP2K4	51.7%	0.003	0.02

Glycan Structures Biosynthesis	MGAT4B; FUT8; CSGLCA-T; GALNT10; HS3ST3A1	50.8%	0.003	0.02

Gap Junction	HTR2C; PRKCA; PRKG1	47.9%	0.005	0.02

Non-Small Cell Lung Cancer	AKT2; PRKCA	42.6%	0.007	0.03

Small Cell Lung Cancer	AKT2; PTK2	35.6%	0.013	0.05

Long-Term Depression	PRKCA; PRKG1	33.3%	0.014	0.05

Insulin Signaling	AKT2; SREBF1	36.0%	0.014	0.05

Long-Term Potentiation	PPP3CA; PRKCA; RPS6KA2	27.1%	0.005	0.06

T-Cell Receptor Signaling	AKT2; PPP3CA	32.3%	0.016	0.09

Wnt Signaling	PPP3CA; PRKCA	21.6%	0.020	0.09

22 out of 74 pathways containing host genes show a significant overrepresentation of targets within the pathway at a FDR of 10%. Host genes that were predicted targets of their own miRNA were removed from the count. Interestingly, the list of pathways contains many pathways crucial for development and signal transduction, or associated with neoplastic transformation.

### Implications for Cancer Pathogenesis

Integration of major KEGG pathway information with expression data from two publicly available datasets [35,36] helped us investigate the idea of loss of negative feedback circuitry.

**Figure 2:**
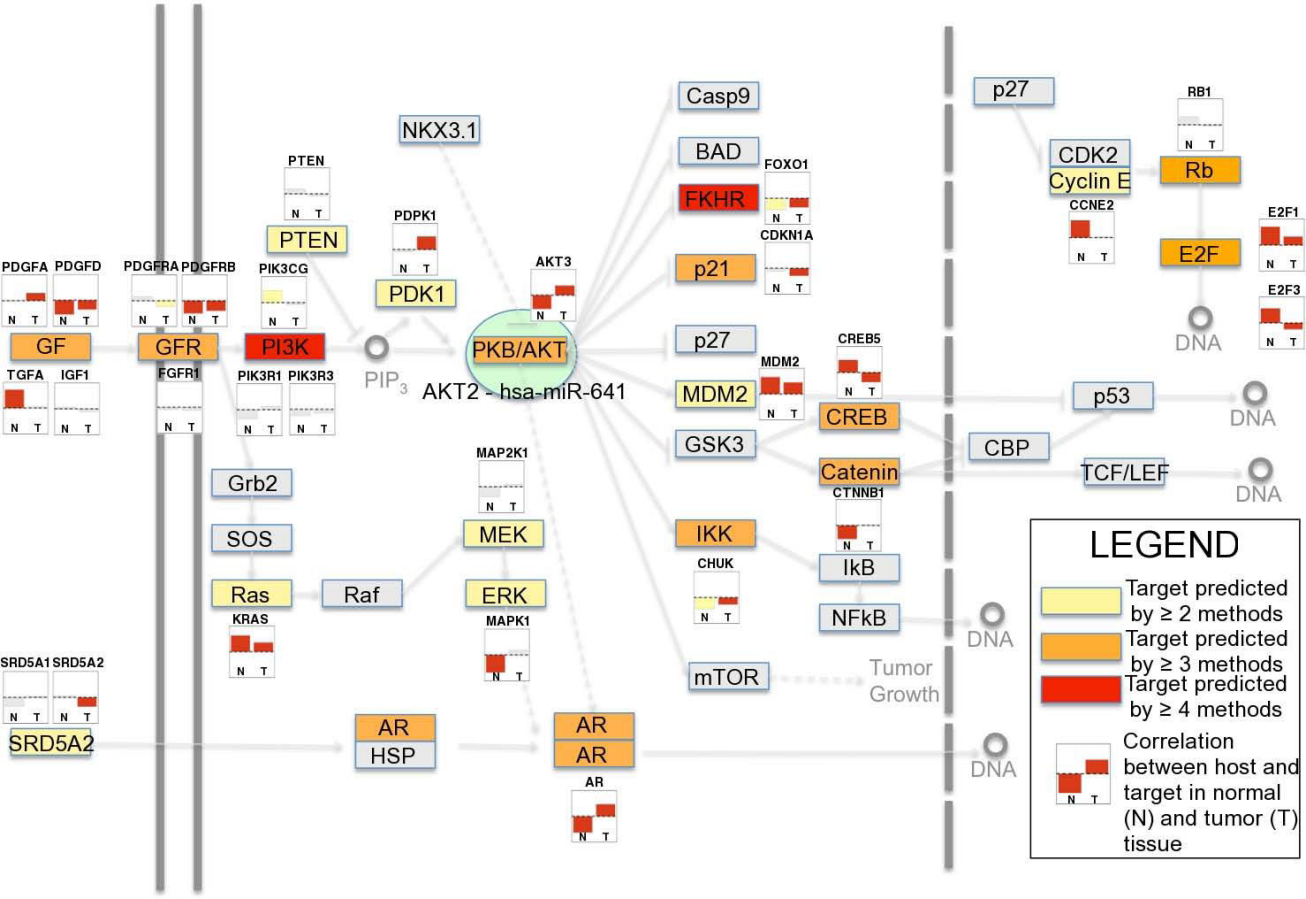
**Correlation of Predicted miRNA Targets with Hosts**. The *PI3K *- *PKB*/*AKT *pathway is believed to be a key component in cancer development. We compared correlation of miRNA predicted targets (prediction agreement ≥ 2) to the respective host in normal (N) and tumor (T) tissue. Several of the hypothesized targets display features predicted by our model, such as *AR*, *PDGFB*, *PDGFRB*, *AKT3 *and *MAPK1*.

KEGG ID "05215 - Prostate Cancer" contains a single known miRNA host (*AKT2*), and it is not predicted to be targeted by its intronic miRNA (*hsa-miR-641*). The correlation between the expressions of host and predicted targets involved in the pathway were calculated. Figure 2 shows a simplified representation based on the KEGG pathway information. Host and corresponding targets are color-coded, where the green oval indicates the host, *AKT2*, and yellow, orange, and red indicate whether two, three or four methods agreed on the target prediction. In line with the hypothesis of an interactome feedback circuitry, predicted targets of *hsa-miR-641 *appear to be in close proximity and in functional synergy with its host. A similar target pattern is displayed by both miRNAs, *hsa-miR-641 *and *hsa-mir-634*, in the non-small-cell lung cancer pathway (additional file 4).

**Figure 3:**
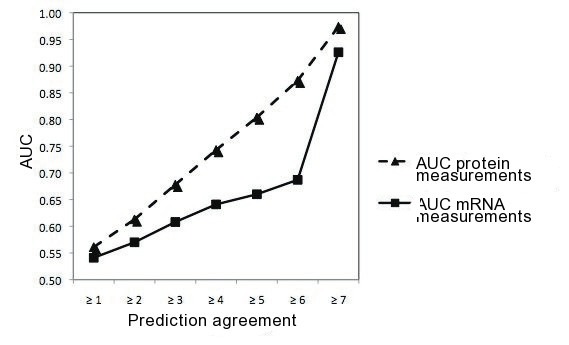
**Prediction Agreement as a Measure of Confidence**. When constructing an ROCs on protein measurements, there is an almost linear relationship of the resulting AUCs and prediction agreement. This is also true for mRNA measurements, though the slope is less steep.

The correlation between host and target expression levels is shown in a two-bar plot. The first bar, labeled "N", represents the correlation between host and target in normal tissue. The second bar, labeled "T", represents the correlation between host and target in cancerous tissue. In the prostate cancer dataset, seven of the fifteen targets are more negatively correlated in healthy tissue than in cancer. In four cases (*AKT3*, *AR*, MAPK1, and *CTNNB1*), we could observe a significant negative correlation in normal tissue, which was either non-significant or was significantly positive in cancer. A similar pattern could be observed in the non small cell lung cancer pathway.

## Discussion and Conclusions

Since the first discovery of miRNAs, our understanding of biogenesis and regulation has exponentially grown. In the recent past, it has been estimated that miRNAs that reside in intronic or exonic regions of other genes may be the dominating class [9]. However, functional aspects of intragenic miRNAs are still largely unknown.

It is generally believed that both host and miRNA share regulatory control [4-6], although a recent study found that transcription of roughly 30% of intragenic miRNAs may be initiated independently [3]. After mapping miRNAs to known genes, we found that most intronic miRNAs are oriented in the same direction as their host gene, significantly more than would be expected by chance. Several hypotheses related to this preferential orientation have been suggested. First, most of intragenic miRNAs may not present their own promoter and be dependent to the transcription of their host gene. Second, miRNAs may present their own promoter, and directional bias may prevent physical interference between RNA polymerases transcribing the host gene and RNA polymerases transcribing the miRNA gene [4].

Baskerville and Bartel identified significant correlation between the expression levels of intronic miRNAs and their host genes, suggesting co-regulation [4]. We furthermore found that more than half of intronic miRNAs are found in the 5' regions of their host genes, where introns are firstly excised. It is well known that transcriptional activity is higher towards the 5' region of a gene [37] and also that regulatory motifs tend to reside in these regions [38]. From a functional perspective, these findings may suggest dependency between host and miRNA transciption. In order to characterize the relationship between intronic miRNAs and their hosts, we identified properties of the set of host genes, as well as a set of high confidence targets. Whereas Golan et al. [39] showed in a recent work that intronic miRNA density is lower in large host genes, we provided evidence that the class of host genes in general is significantly longer and contains more and larger introns. This increases transcriptional efforts for the cell and is considered a characteristic of tightly regulated genes [40]. Interestingly, these features can also be found in a high-confidence set of targets (i.e. prediction agreement ≥ 6 methods), which may support the idea of miRNAs as regulators of their own host genes. Additionally, the 3'-UTRs of host genes predicted to be targeted by their own miRNA are significantly longer, exposing the message to more regulatory control mechanisms, such as targeting by miRNAs or ARE mediated mRNA decay. Interestingly, host genes contain significantly more AREs. Many of these properties have been shown to be features of proto-oncogenes and the sum of these findings may suggest tight regulatory control of these genes [21,23,41]. Surveying GOBP and KEGG pathways, we found host genes to be associated with metabolic, biosynthetic, gene regulative processes, and signaling pathways. These categories capture major functional aspects of miRNAs in general, as is reflected by miRNA involvement in diseases such as cancer [32], muscle disorders [27], or neurodegenerative diseases [42]. We then assessed predicted targets, using agreement between six distinct prediction algorithms and a database of validated miRNA targets as a measure of confidence. First, we identified 56 miRNAs predicted to target their own host. Interestingly, more of these miRNA-host gene pairs are conserved than of the remaining miRNA-host gene pairs (Table 4). Recently, Sun et al. validated the predicted interaction between *hsa-miR-126 *and its host EGFL7 [43,44]. By integrating KEGG pathways with these predictions, we observed for 22 of the 74 pathways that host genes were associated with a higher number of targets within the pathway than would be expected by chance alone. A visual representation of the targets of *AKT2*'s intronic miRNA *hsa-miR-641*, for example, showed how components of many protein complexes involved in the signal transduction of growth factor signalling may be potential targets of *hsa-miR-641 *(Figure 2). The combination of these findings indicates that intragenic miRNAs may play a role in interactome feedback circuitries, as visualized in Figure 1d, as an additional security switch for genes requiring narrow control. A subset of up to 20% of intragenic miRNAs may directly regulate the host expression (we referred to this phenomenon as "first-order feedback"). Moreover, intragenic miRNAs display targeting patterns that appear not only to influence their hosts' expression levels, but also their functional environment. The observation that structural properties of a set of high-confidence-prediction target genes, such as long 3'-UTRs, length, and number of AREs, resemble those of host genes emphasize the concept of regulation of interacting gene products in highly restricted settings.

**Table 4:** Conservation of miRNA-Host Pairs

Organism	miRNA-Host Pairs Predicted to Target own Host		miRNA-Host Pairs Not Predicted to Target own Host		p-Value
		
	Conserved	Total	Conserved	Total	
*Homo sapiens - Mus musculus*	18 (35.2%)	51	41 (24.5%)	167	0.18

*Homo sapiens - Canis familiaris*	1 (2.12%)	47	0	142	0.56

*Homo sapiens - Gallus gallus*	5 (12.5%)	40	1 (0.71%)	139	0.001

The subset of intragenic miRNA host pairs where the miRNA is predicted to target its own host shows a tendency to be more conserved. However, statistical significance can only be shown for conservation between human and chicken (2-sample test for equality of proportions with continuity correction).

Loss of negative feedback control systems is a well-known mechanism by which cancer develops. Blenkiron and coworkers [11] recently suggested that miRNA processing might be disturbed in cancer. If expression levels of intragenic miRNAs are reduced, as observed by some authors [13,45], subsequently important signalling pathways may lose inhibition and this may facilitate uncontrolled cell growth. In a recent study, Tavazoie et al. analyzed six miRNAs that were significantly under-expressed in breast cancer LM2 cells, as compared to normal breast tissue. Four of these miRNAs were intragenic [46]. The authors reported that loss of the intronic miRNA *hsa-miR-335*, which resides in intron 2 of its host gene *MEST*, led to increased migration and invasion rates and hence increased metastatic capacity. Additionally, they could show that *hsa-miR-126 *(intron 7, host *EGFL7*) significantly reduced proliferation of breast cancer cells. Likewise, *hsa-miR-151 *has been shown to be downregulated in chronic myloid leukemia through *BCR/ABL *[47], and silencing its host gene *PTK2 *inhibits leukemogenesis [48]. A similar pattern can for example be found for *hsa-miR-504 *and *FGF13 *[49,50].

Changes in miRNA biosynthesis such as those found in cancer can interfere with the coordination of expression of miRNA and host. Thus, a negative correlation between expression levels of host and genes targeted by its intragenic miRNA in normal tissue (given that the host is not targeted by the miRNA it contains) and a less negative or even positive correlation in cancerous tissue might be expected. This phenomenon was observed in two distinct datasets in different malignancies (Figure 2, additional file 4 and additional file 6). A key to pathogenesis of both entities is the phosphatidylinositol 3-kinase(PIK3)/AKT signaling pathway, deregulation of which has been reported in several cancers, including prostate cancer [51], lung cancer [52], ovarian cancer [53,54], breast cancer [53,55], and colon tumors [54]. Whereas Noske et al. discovered that silencing *AKT2 *through RNA interference leads to reduction in ovarian cancer cell proliferation [56], Maroulakou and coworkers reported accelerated development of polyoma middle T and ErbB2/Neu-driven mammary adenocarcinomas in mice after *AKT2 *ablation [57]. Although these findings would appear to be contradictory at first, they can be explained by an intragenic miRNA-driven negative regulatory loop that is disturbed in cancer. Whereas in the first experiment *AKT2 *was targeted on mRNA level (and therefore mimicking the role of the corresponding intronic miRNA), in the second experiment both host mRNA and miRNA (if it exists in mouse) were downregulated, and therefore may have disabled a potential negative feedback regulation by *hsa-miR-641*.

One must remember, however, that regulatory networks are far more complex in reality than what we are currently able to model. Transcription factors, enhancers, silencers, and epigenetic modifications play major roles in cancer development and may influence correlation among expression levels of hosts and targets. Also, target prediction methods are error prone, and at this point we can only speculate about the true nature of events and therefore plan to conduct further experiments in which the hypotheses presented can be tested. For example, by integrating different target prediction methods, roughly 20% of intragenic miRNAs were predicted to target their own host. Though this number is significantly higher than expected by chance, it still does not cover the majority of miRNAs. For one, this number may underestimate the true number of miRNAs targeting their own host due to limitations of target prediction methods. Additionally, it has lately been shown that transcription of one third of intronic miRNAs can be initiated independently of the host's transcription [3], in which case direct feedback cannot be claimed. Also, we only investigated feedback on the level of direct miRNA-host interaction and on the order of the interactome based on the KEGG database. However, knowledge about interaction of proteins is still limited and cotranscription of host and miRNA may enable more complex mechanisms. Limitations to current knowledge may also justify, why a significant fraction of predicted targets in do not show the expected behaviour. Indeed, *Cyclin E *and *E2F *in Figure 2 show opposite behavior than what we would expect. Neither of these genes might actually be a target of *hsa-miR-641*; there may also exist stronger regulating elements that control their expression, or the primary mode of silencing in that specific situation may be through translational repression. Nevertheless, it is interesting how key molecules in two different datasets displayed predicted correlation patterns.

Further experiments and biological validation of computational evidence presented here may have great implications, especially in cancer therapy. Modern therapies usually target central molecules, such as *AKT *and *PI3K *with some success. However, these techniques control only single elements in a cascade of complex signalling events. In summary, our findings encourage more focused research on intragenic miRNAs and their targets.

## Methods

### Classification of miRNAs

miRNA genomic coordinates from miRBase release 11 (April 2008) [58-60] were crossed to genomic coordinates of RNA Reference Sequences (http://www.ncbi.nlm.nih.gov/RefSeq; Release 31) [20] downloaded from UCSC Genome Browser http://genome.ucsc.edu. To each genomic mapped RefSeq sequence, a single gene was assigned. The subset of miRNAs whose coordinates mapped to an annotated gene was defined as intragenic. Intragenic miRNAs were classified as exonic when their coordinates overlapped with any observed exonic region, and intronic otherwise.

### Host Genes' Intronic miRNA Distribution

Introns were sequentially enumerated based on gene orientation. For each intron number, host genes containing miRNAs in this intron were counted. We calculated the expected number of genes containing an intronic miRNAs in a given intron number by adding all intron lengths of introns with the respective intron number and dividing it by the summed length of all host genes' introns, thus accounting for intron frequency and length.

### Gene Ontology

The Gene Ontology [61] classifications of all 246 host genes of intragenic miRNA genes that were located on the same strand as their host gene were surveyed using Cytoscape 2.6.0 [62] and BiNGO 2.3 [63]. We focused our attention on those categories that were disproportionately overrepresented. The setting "Hypergeometric test" was chosen to calculate the probability of observing an equal or greater number of genes in a given functional category than in the test set. The False Discovery Rate (FDR), which is the standard setting in BiNGO 2.3 [63], was controlled.

### Pathways identification

The statistical programming software R 2.7.1 was used in combination with Bioconductor [64,65] packages AnnBuilder 1.18.0, KEGG.db version 2.2.0, and GOStats version 1.7.4 to acquire a list of pathways that were associated with one or more of the 246 host gene proteins.

### Target Predictions

Strategies to perform high-throughput miRNA target validation are still very limited. Therefore, target prediction algorithms are employed to allow large-scale assessment of miRNA-target interaction. However, usage of target prediction methods raises two difficulties. First, target prediction methods are known to suffer from a significant number of false positive predictions. We reasoned that a possible way to address this problem would be to estimate statistical significance by generating background distributions by the very same methods. Hence, if target predictions were too close to random, the mean of the generated background distribution should be close to the observed number, whereas a significant finding should not be affected by the absolute number of false positives. Second, different target prediction algorithms incorporate different types of information about miRNA target interactions. To overcome individual biases that may be introduced by one specific method and use the wide range of experimental knowledge gained, we integrated predictions from six current algorithms. Precalculated target predictions for TargetScan release 4.2 [66] (April 2008), PITA [67] catalog version 6 (August 2008), MirTarget2 (mirDB) version 2.0 [68,69] (December 2007), miRanda [70] (September 2008), RNA22 [71] (November 2006) and PicTar 5-way [72] were downloaded. We also included TarBase version 5.0c [73] (June 2008) as a reference database for miRNA target interactions with published evidence; only targets with a "Support Type" value of either "True" or "Microarray" were selected. Some miRNA symbols did not exactly match entries in the database for various reasons, including use of non-official names or older miRBase releases. Whenever a miRNA symbol could not be found, matching was attempted to an extension such as "-1" or "a" (for example, *hsa-mir-511 *in mirTarget2 was matched to *hsa-mir-511-1 *and *hsa-mir-511-2*). If the miRNA symbol ended with a letter, it was removed to check for other matches (from the PicTar prediction list *hsa-mir-128a *matched to *hsa-mir-128-1*, *hsa-mir-128-2*, and *hsa-mir-128-3 *for example). Predictions for a miRNA symbol were ignored if no matches could be found. Due to the diversity of underlying principles, assumptions, and scoring systems, we defined the prediction agreement, i.e. the number of methods that agree on a certain miRNA target prediction, as a measure of confidence in the target prediction. In recent work, Selbach et al. measured changes in protein and mRNA expression after transfection and overexpression of five different miRNAs (hsa-miR-1, hsa-miR-16, hsa-miR-30a, hsa-miR-255, hsa-let-7b) in HeLa cells [74]. We evaluated the different target prediction methods used in this study by measuring the abundance of predicted products (mRNA or the proteins encoded by these mRNAs, a continuous value) and assessing discrimination by areas under the ROC curve using the predicted targets as the binary outcome. All five miRNA datasets were pooled (see additional file 3 for details). The AUC (Area under Receiver Operator Characteristic (ROC) Curve) measures how well predictions and non-predictions can be discriminated at all possible thresholds, with a value of 0.5 indicating no discrimination and a value of 1 indicating perfect discrimination. Target prediction methods varied greatly in AUCs, ranging from 0.55 to 0.92 in protein measurements. With increasing prediction agreement, an almost linear increase in AUC can be observed, indicating that prediction agreement may be used as a proxy for the confidence of a predicted miRNA target interaction (Figure 3.

### Gene Expression Datasets

Two publicly available mRNA expression datasets (GSE6956, GSE7670) were downloaded from the Gene Expression Omnibus http://www.ncbi.nlm.nih.gov/geo. We included 87 prostate samples (69 tumor and 18 healthy tissue samples) [35] and 60 lung samples (31 non-small-cell lung cancer and 29 healthy lung tissue samples) [36]. Preprocessing was carried out using BioConductor packages [64,65]. Data from protein and mRNA expression change after miRNA transfection experiments were downloaded from http://psilac.mdc-berlin.de[74].

### Genomic Host and Target Gene Properties

In order to assess genomic properties of host genes (n = 246), we constructed a set of control genes (n = 2460) that would match chromosome and strand distribution of host genes in order to exclude structural differences due to chromosomal specificities. We defined miRNA target interactions predicted by at least 6 methods as "high confidence targets" (n = 326). These predictions cover 33 host genes and 43 miRNAs when at least six methods are required and 239 hosts and 272 miRNAs when at least 2 methods are required. Statistical testing was done using Mann-Whitney-U test. For the analysis of total length and 3'-UTR length of host genes, hosts were additionally split into two groups, dependent on whether they were predicted to be targets of their intragenic miRNA. We combined the Kruskal-Wallis rank sum test with post-hoc pairwise Mann-Whitney-U test with Bonferroni correction (p < 0.016 defined as significance cut-off for three pairwise comparisons). Assessment of host genes predicted to be targeted by their intragenic miRNAs was carried out as follows: Out of the 2460 control genes, we sampled 1000 sets of genes of size 246 that would match the host gene 3'-UTR length distribution (no significant difference in Mann-Whitney-U test). Intragenic miRNAs were assigned to genes in the sets and the number of genes predicted to be targeted by that miRNA was calculated. Similarly, the observed number of miRNAs predicted to target their own host was assessed by exchanging host genes for randomly chosen genes from predicted targets and recalculation of the number of miRNAs predicted to target their host. Robustness of this approach was tested by additionally requiring a vote from each of two groups of three prediction methods each.

### Host-miRNA Conservation

HomoloGene database NCBI release 61 http://www.ncbi.nlm.nih.gov/homologene and mirBase release 11 (April 2008) [58-60] were used to identify homologous host genes in *Homo sapiens*, *Canis familiaris*, and *Gallus gallus*. Proportions of conserved miRNA-host gene pairs for miRNAs predicted and miRNAs not predicted to target their own host were calculated. Similarly, we used information on target site conservation from TargetScan to calculate the proportion of conserved targetsites of predicted target host interactions in the hosts' pathway and of those not in the hosts' pathway. Statistical significance was assessed using the 2-sample test for equality of proportions with continuity correction.

### Target Coverage

The union of predicted targets included more than 90% of all known human genes. Since target prediction methods are very different, they are difficult to compare. In this work, only targets that were predicted by at least two different methods were considered in the calculation of target coverage. This reduced the total number of predictions by almost 70%.

We defined the set *S_p _*as the set of genes linked to a pathway and *S_t _*as the set of predicted targets of the miRNAs associated with the pathway through their host genes. The target coverage (*C*) for a pathway was defined as

$$C=\frac{\left|S_{p}\cap S_{t}\right|}{\left|S_{p}\right|}.$$

Statistical significance of target enrichment within a pathway was tested by randomly sampling |*S_p_*| genes from a universe of all known genes, replacing the genes within the pathway with the set of genes in the random sample (*S_i_*), and subsequently calculating a new "random" target coverage *C_i_'*. This procedure was repeated 1000 times, allowing estimation of the probability as the number of times a target coverage *C_i_' *greater or equal to *C *was observed. We defined the indicator function I(*C_i_',C*) as

$$I({C^{\prime }}_{i},C)\{\begin{array}{cc} 1 & if\;C_{i}{}^{\prime }\geq C\\ 0 & otherwise \end{array}.$$

Hence, the probability of observing greater or equal target coverage for a given pathway could be estimated as

$$p(C^{\prime }\geq C)=\frac{\sum_{i=1}^{1000}I\left(\frac{\left|S_{i}\cap S_{t}\right|}{\left|S_{i}\right|},C\right)}{1000},\text{ where }|S_{i}|=|S_{p}|.$$

Analogously, the enrichment statistics for miRNAs targeting their own hosts were calculated, where *S_p _*was defined as the set of host genes, *S_t _*as the set of targets of the intragenic miRNAs of these host genes, and *S_i _*as the set of |*S_p_*| randomly sampled genes (out of the non-redundant set of predicted targets for these miRNAs). The R-package '*q-value' *was used to account for multiple hypothesis testing by controlling the False Discovery Rate (FDR) to be < 10%.

## Authors' contributions

LCGH contributed by developing the design of the study, performing bioinformatics analyses, and writing the paper.

PAFG contributed by developing the design of the study and performing bioinformatics analyses.

WPK contributed by providing background knowledge and writing the paper.

LOM contributed by developing the design of the study, mentoring the project and writing the paper.

All authors have read and approved the final manuscript.

## Supplementary Material

Additional file 1 **Additional Information on Intragenic miRNAs**. The table in additional file 1 contains information on intragenic miRNAs, such as genomic position, name and RefSeq ID of the host gene, and orientation. Click here for file

Additional file 2 **Distribution of intragenic miRNAs**. Additional file 2 contains an additional barplot showing the distribution of intronic miRNAs across their hosts' introns, as well as a theoretically expected distribution taking intron frequency and size into consideration. The first figure on page 1 shows a barplot of expected and observed distribution of intragenic miRNAs across their hosts' interactome. The second figure is a repetition of Figure 1b, for better comparison. The second page contains the underlying data in table format. Click here for file

Additional file 3 **Evaluation of Target Prediction Methods**. Based on protein and mRNA expression measurements in miRNA transfection experiments, we evaluated the target prediction methods used in this study, as well as prediction agreement as a method of its own. We estimated sensitivity, specificity, and AUC for target prediction methods used and prediction agreement based on the Selbach data [74] for changes in mRNA and protein expression after miRNA overexpression. Click here for file

Additional file 4 **Non Small Cell Lung Cancer**. The figure is analogous to Figure 2, for a non small cell lung cancer mRNA expression microarray dataset. Click here for file

Additional file 5 **Full Pathway Information**. The table provides all 74 KEGG pathways associated with one or more host genes. For each KEGG pathway KEGG ID, pathway name, p-value and odds ratio for the observed number of host genes, total number of expected genes, total number of observed genes, total number of genes in that pathway, pathway url, host gene names and Entrez-IDs, target coverage and p-value, proportion of targets with conserved target sites within the hosts' pathway, proportion of targets with conserved target sites not within the hosts' pathway, q-value of the difference of these two proportions, and Entrez gene IDs for all predicted targets are provided. The asterisk behind a gene ID indicates a conserved target site for that target. It is important to note, however, that a proportion calculated from these gene IDs may differ from the proportion given, as the gene IDs are based on agreement of two prediction methods, whereas the proportion of conserved targets was calculated on predictions made by targetscan only. Click here for file

Additional file 6 **Additional file **6** contains correlation data from which Figure **2** and additional file **4** have been generated**. For both pathways, hosts and their predicted targets as well as correlation and p-value are provided. Click here for file
