# Supplementary material for: A potential role for intragenic miRNAs on their hosts' interactome
Source: BMC Genomics. 2010 Oct 1;11:533. doi: 10.1186/1471-2164-11-533 (PMC3091682; doi:10.1186/1471-2164-11-533)
Supplement: Additional file 1 — Additional Information on Intragenic miRNAs. The table in additional file 1 contains information on intragenic miRNAs, such as genomic position, name and RefSeq ID of the host gene, and orientation. [file 1471-2164-11-533-S1.PDF]

MiRNA Accession ID	Symbol	Chromosome	Strand	Start	End	Exonic	Hostname	RefSeq ID	Intron/Exon	Intron Length
MI0003556	hsa-mir-551a	1	-	3467119	3467214	No	MEGF6	NM_001409	4	55577
MI0006352	hsa-mir-1290	1	-	19096152	19096229	No	ALDH4A1	NM_170726	1	12355
MI0006390	hsa-mir-1256	1	-	21187394	21187512	No	EIF4G3	NM_003760	5	21484
MI0000749	hsa-mir-30e	1	+	40992614	40992705	No	NFYC	NM_014223	5	4873
MI0000736	hsa-mir-30c-1	1	+	40995543	40995631	No	NFYC	NM_014223	5	4873
MI0006397	hsa-mir-1262	1	-	68421789	68421881	No	GPR177	NM_001002292	2	34706
MI0000483	hsa-mir-186	1	-	71305902	71305987	No	ZRANB2	NM_005455	8	2340
MI0003558	hsa-mir-553	1	+	100519385	100519452	No	RTCD1	NM_001130841	9	7910
MI0005767	hsa-mir-942	1	+	117438788	117438873	No	TTF2	NM_003594	18	2406
MI0003559	hsa-mir-554	1	+	149784896	149784991	No	TUFT1	NM_020127	1	21663
MI0003561	hsa-mir-555	1	-	153582765	153582860	Yes	ASH1L	NM_018489	21	N/A
MI0000466	hsa-mir-9-1	1	-	154656757	154656845	No	C1orf61	NM_006365	2	9932
MI0005116	hsa-mir-765	1	-	155172547	155172660	No	ARHGEF11	NM_198236	40	756
MI0003562	hsa-mir-556	1	+	160578960	160579054	No	NOS1AP	NM_014697	5	10708
MI0005713	hsa-mir-921	1	-	164390604	164390659	No	FAM78B	NM_001017961	2	95221
MI0006436	hsa-mir-1255b-2	1	+	166234522	166234588	No	IQWD1	NM_018442	7	9040
MI0006357	hsa-mir-1295	1	-	169337493	169337571	No	FMO3	NM_006894	7	10993
MI0000290	hsa-mir-214	1	-	170374561	170374670	No	DNM3	NM_015569	7	122283
MI0000281	hsa-mir-199a-2	1	-	170380298	170380407	No	DNM3	NM_015569	7	122283
MI0003123	hsa-mir-488	1	-	175265122	175265204	No	ASTN1	NM_004319	5	4900
MI0006425	hsa-mir-1278	1	+	191372256	191372336	No	CDC73	NM_024529	5	2494
MI0006321	hsa-mir-1231	1	+	200044362	200044453	No	NAV1	NM_020443	18	91
MI0000285	hsa-mir-205	1	+	207672101	207672210	Yes	LOC642587	NM_001104548	4	N/A
MI0000291	hsa-mir-215	1	-	218357818	218357927	No	IARS2	NM_018060	11	10761
MI0000488	hsa-mir-194-1	1	-	218358122	218358206	No	IARS2	NM_018060	11	10761
MI0006442	hsa-mir-664	1	-	218440503	218440584	No	RAB3GAP2	NM_012414	9	5876
MI0003839	hsa-mir-320b-2	1	-	222511329	222511466	No	NVL	NM_002533	18	17714
MI0006275	hsa-mir-1182	1	-	229222197	229222293	Yes	FAM89A	NM_198552	2	N/A
MI0007258	hsa-mir-1537	1	-	234082923	234082983	No	LYST	NM_000081	1	33174
MI0003815	hsa-mir-1301	2	-	25405013	25405094	No	DNMT3A	NM_175629	1	28267
MI0003564	hsa-mir-558	2	+	32610724	32610817	No	BIRC6	NM_016252	61	11688
MI0003565	hsa-mir-559	2	+	47458318	47458413	No	TACSTD1	NM_002354	5	1874
MI0000447	hsa-mir-128-1	2	+	136139437	136139518	No	R3HDM1	NM_015361	18	13937
MI0005755	hsa-mir-933	2	-	175740607	175740683	No	ATF2	NM_001880	1	16890
MI0006392	hsa-mir-1258	2	-	180433808	180433880	No	ZNF385B	NM_152520	1	90427
MI0003567	hsa-mir-561	2	+	188870464	188870560	No	GULP1	NM_016315	1	90649
MI0006380	hsa-mir-1245	2	+	189551063	189551132	No	COL3A1	NM_000090	1	10190
MI0006375	hsa-mir-548f-2	2	-	212999232	212999329	No	ERBB4	NM_005235	1	413543
MI0000084	hsa-mir-26b	2	+	218975613	218975689	No	CTDSP1	NM_021198	4	628
MI0000463	hsa-mir-153-1	2	-	219867077	219867166	No	PTPRN	NM_002846	19	3450
MI0006379	hsa-mir-1244	2	+	232286268	232286352	Yes	PTMA	NM_002823	5	N/A
MI0003568	hsa-mir-562	2	+	232745607	232745701	No	DIS3L2	NM_152383	9	46692
MI0000478	hsa-mir-149	2	+	241044091	241044179	No	GPC1	NM_002081	1	22944
MI0005560	hsa-mir-885	3	-	10411173	10411246	No	ATP2B2	NM_001001331	5	12549
MI0000727	hsa-mir-128-2	3	+	35760972	35761055	No	ARPP-21	NM_016300	18	48415
MI0000083	hsa-mir-26a-1	3	+	37985899	37985975	No	CTDSPL	NM_001008392	5	3523
MI0006313	hsa-mir-1226	3	+	47866049	47866123	No	DHX30	NM_014966	21	74
MI0001448	hsa-mir-425	3	-	49032585	49032671	No	DALRD3	NM_018114	1	2526
MI0000465	hsa-mir-191	3	-	49033055	49033146	No	DALRD3	NM_018114	1	2526
MI0003572	hsa-mir-566	3	+	50185763	50185856	No	SEMA3F	NM_004186	2	14057
MI0000433	hsa-let-7g	3	-	52277334	52277417	No	WDR82	NM_025222	2	3692
MI0006431	hsa-mir-1284	3	-	71673811	71673930	No	FOXP1	NM_032682	2	87992
MI0003573	hsa-mir-567	3	+	113314338	113314435	No	C3orf52	NM_024616	4	3349
MI0000240	hsa-mir-198	3	-	121597205	121597266	Yes	FSTL1	NM_007085	11	N/A
MI0006437	hsa-mir-1280	3	+	129563698	129563791	No	EEFSEC	NM_021937	6	49694
MI0000438	hsa-mir-15b	3	+	161605070	161605167	No	SMC4	NM_001002800	5	7414
MI0000115	hsa-mir-16-2	3	+	161605227	161605307	No	SMC4	NM_001002800	5	7414
MI0003576	hsa-mir-569	3	-	172307147	172307242	No	TNIK	NM_015028	21	5548
MI0006383	hsa-mir-1248	3	+	187987155	187987260	No	EIF4A2	NM_001967	7	480
MI0000086	hsa-mir-28	3	+	189889263	189889348	No	LPP	NM_005578	6	98421
MI0005769	hsa-mir-944	3	+	191030405	191030492	No	TP63	NM_003722	4	55704
MI0005714	hsa-mir-922	3	-	198885764	198885844	Yes	KIAA0226	NM_014687	20	N/A
MI0003578	hsa-mir-571	4	+	333946	334041	No	ZNF141	NM_003441	3	28232
MI0005768	hsa-mir-943	4	-	1957909	1958002	Yes	WHSC2	NM_005663	5	N/A
MI0000097	hsa-mir-95	4	-	8057928	8058008	No	ABLIM2	NM_001130083	16	15130
MI0000294	hsa-mir-218-1	4	+	20138996	20139105	No	SLIT2	NM_004787	15	3775
MI0003581	hsa-mir-574	4	+	38546048	38546143	No	FAM114A1	NM_138389	1	562
MI0003582	hsa-mir-575	4	-	83893514	83893607	No	SCD5	NM_001037582	1	92891
MI0006389	hsa-mir-1255a	4	-	102470482	102470594	No	PPP3CA	NM_000944	1	150621
MI0003583	hsa-mir-576	4	+	110629303	110629400	No	SEC24B	NM_006323	4	9544
MI0000775	hsa-mir-367	4	-	113788479	113788546	No	LARP7	NM_016648	5	1699
MI0000774	hsa-mir-302d	4	-	113788609	113788676	No	LARP7	NM_016648	5	1699
MI0000738	hsa-mir-302a	4	-	113788788	113788856	No	LARP7	NM_016648	5	1699
MI0000773	hsa-mir-302c	4	-	113788968	113789035	No	LARP7	NM_016648	5	1699
MI0000772	hsa-mir-302b	4	-	113789090	113789162	No	LARP7	NM_016648	5	1699
MI0006373	hsa-mir-1243	4	+	114247468	114247560	No	ANK2	NM_001127493	2	269900
MI0003584	hsa-mir-577	4	+	115797364	115797459	No	UGT8	NM_001128174	2	40291
MI0003585	hsa-mir-578	4	+	166526844	166526939	No	CPE	NM_001873	1	84860
MI0005562	hsa-mir-887	5	+	15988291	15988369	No	FBXL7	NM_012304	3	7947
MI0003586	hsa-mir-579	5	-	32430241	32430338	No	ZFR	NM_016107	11	4720
MI0003587	hsa-mir-580	5	-	36183751	36183847	No	LMBRD2	NM_001007527	1	8148
MI0006410	hsa-mir-1274a	5	+	41511491	41511561	No	PLCXD3	NM_001005473	2	127888

MI0003588	hsa-mir-581	5	-	53283091	53283186	No	*ARL15*	NM_019087	4	226487
MI0001648	hsa-mir-449a	5	-	54502117	54502207	No	*CDC20B*	NM_152623	2	25730
MI0003673	hsa-mir-449b	5	-	54502231	54502327	No	*CDC20B*	NM_152623	2	25730
MI0003589	hsa-mir-582	5	-	59035189	59035286	No	*PDE4D*	NM_001104631	1	677199
MI0006420	hsa-mir-548p	5	-	100180085	100180168	No	*ST8SIA4*	NM_005668	4	43972
MI0006376	hsa-mir-548f-3	5	-	109877429	109877515	No	*FLJ43080*	NM_001039763	11	143529
MI0006379	hsa-mir-1244	5	+	118338180	118338264	No	*DTWD2*	NM_173666	5	43643
MI0006351	hsa-mir-1289-2	5	-	132791187	132791297	No	*FSTL4*	NM_015082	3	166197
MI0005532	hsa-mir-874	5	-	137011160	137011237	No	*KLHL3*	NM_017415	8	18152
MI0003591	hsa-mir-584	5	-	148422069	148422165	No	*SH3TC2*	NM_024577	1	10729
MI0000786	hsa-mir-378	5	+	149092581	149092646	No	*PPARGC1B*	NM_133263	1	90011
MI0006356	hsa-mir-1294	5	+	153706859	153707000	No	*GALNT10*	NM_198321	4	46537
MI0000109	hsa-mir-103-1	5	-	167920479	167920556	No	*PANK3*	NM_024594	5	2234
MI0007261	hsa-mir-103-1-as	5	+	167920487	167920548	No	*PANK3*	NM_024594	2	2234
MI0000295	hsa-mir-218-2	5	-	168127729	168127838	No	*SLIT3*	NM_003062	14	10090
MI0003592	hsa-mir-585	5	-	168623183	168623276	No	*SLIT3*	NM_003062	1	49052
MI0003814	hsa-mir-1271	5	+	175727555	175727640	No	*ARL10*	NM_173664	2	2224
MI0006319	hsa-mir-1229	5	-	179157884	179157952	No	*MGAT4B*	NM_014275	13	68
MI0000802	hsa-mir-340	5	-	179374909	179375003	No	*RNF130*	NM_018434	2	27140
MI0005561	hsa-mir-877	6	+	30660088	30660173	No	*ABCF1*	NM_001025091	13	85
MI0006326	hsa-mir-1236	6	-	32032595	32032696	No	*RDBP*	NM_002904	3	101
MI0003594	hsa-mir-586	6	-	45273389	45273485	No	*SUPT3H*	NM_181356	4	215788
MI0003596	hsa-mir-548b	6	-	119431911	119432007	No	*FAM184A*	NM_024581	1	53326
MI0008334	hsa-mir-1913	6	-	166842832	166842911	No	*RPS6KA2*	NM_001006932	5	1993
MI0000815	hsa-mir-339	7	-	1029095	1029188	No	*C7orf50*	NM_001134395	2	117112
MI0003599	hsa-mir-589	7	-	5501976	5502074	No	*FBXL18*	NM_024963	3	9037
MI0006276	hsa-mir-1183	7	+	21477201	21477289	No	*SP4*	NM_003112	3	46234
MI0003600	hsa-mir-550-1	7	+	30295935	30296031	No	*ZNRF2*	NM_147128	1	37814
MI0006399	hsa-mir-548n	7	-	34946897	34946971	No	*DPY19L1*	NM_015283	18	1436
MI0006332	hsa-mir-1200	7	-	36925487	36925562	No	*ELMO1*	NM_014800	16	118280
MI0003602	hsa-mir-590	7	+	73243464	73243560	No	*EIF4H*	NM_022170	5	4433
MI0006346	hsa-mir-1285-1	7	-	91671265	91671348	No	*KRIT1*	NM_194456	18	11770
MI0003674	hsa-mir-653	7	-	92950008	92950103	No	*CALCR*	NM_001742	2	7422
MI0003124	hsa-mir-489	7	-	92951184	92951267	No	*CALCR*	NM_001742	2	7422
MI0003603	hsa-mir-591	7	-	95686910	95687004	No	*SLC25A13*	NM_014251	4	25823
MI0000082	hsa-mir-25	7	-	99529119	99529202	No	*MCM7*	NM_005916	13	770
MI0000095	hsa-mir-93	7	-	99529327	99529406	No	*MCM7*	NM_005916	13	770
MI0000734	hsa-mir-106b	7	-	99529552	99529633	No	*MCM7*	NM_005916	13	770
MI0006402	hsa-mir-548o	7	-	101833194	101833307	No	*PRKRIP1*	NM_024653	2	2749
MI0003604	hsa-mir-592	7	-	126485378	126485474	No	*GRM8*	NM_001127323	3	201811
MI0003605	hsa-mir-593	7	+	127509149	127509248	No	*SND1*	NM_014390	18	3221
MI0000816	hsa-mir-335	7	+	129923188	129923281	No	*MEST*	NM_177524	2	1632
MI0003125	hsa-mir-490	7	+	136238454	136238581	No	*CHRM2*	NM_001006626	2	81583
MI0006377	hsa-mir-548f-4	7	-	146706042	146706146	No	*CNTNAP2*	NM_014141	15	95317
MI0003760	hsa-mir-671	7	+	150566440	150566557	Yes	*CSGLCA-T*	NM_019015	4	N/A
MI0000464	hsa-mir-153-2	7	-	157059789	157059875	No	*PTPRN2*	NM_002847	19	5118
MI0003607	hsa-mir-595	7	-	158018171	158018266	No	*PTPRN2*	NM_002847	1	97771
MI0003609	hsa-mir-597	8	+	9636592	9636688	No	*TNKS*	NM_003747	17	12536
MI0006653	hsa-mir-1322	8	-	10720293	10720363	No	*PINX1*	NM_017884	5	5881
MI0003610	hsa-mir-598	8	-	10930126	10930222	No	*XKR6*	NM_173683	1	275743
MI0000791	hsa-mir-383	8	-	14755318	14755390	No	*SGCZ*	NM_139167	1	682657
MI0002470	hsa-mir-486	8	-	41637116	41637183	No	*ANK1*	NM_000037	42	5674
MI0003611	hsa-mir-599	8	-	100618040	100618134	No	*VPS13B*	NM_017890	32	35438
MI0005541	hsa-mir-875	8	-	100618190	100618265	No	*VPS13B*	NM_017890	32	35438
MI0006409	hsa-mir-1273	8	-	101105386	101105488	No	*RGS22*	NM_015668	14	30360
MI0003668	hsa-mir-548d-1	8	-	124429455	124429551	No	*ATAD2*	NM_014109	15	775
MI0000809	hsa-mir-151	8	-	141811845	141811934	No	*PTK2*	NM_005607	22	17540
MI0005759	hsa-mir-937	8	-	144967115	144967200	Yes	*SCRIB*	NM_182706	8	N/A
MI0003669	hsa-mir-661	8	-	145091347	145091435	No	*PLEC1*	NM_000445	1	36483
MI0005761	hsa-mir-939	8	-	145590172	145590253	Yes	*CPSF1*	NM_013291	34	N/A
MI0006324	hsa-mir-1234	8	-	145596284	145596367	No	*CPSF1*	NM_013291	9	83
MI0000739	hsa-mir-101-2	9	+	4840297	4840375	No	*RCL1*	NM_005772	8	10573
MI0003126	hsa-mir-491	9	+	20706104	20706187	No	*KIAA1797*	NM_017794	4	2382
MI0000284	hsa-mir-204	9	-	72614711	72614820	No	*TRPM3*	NM_024971	6	43566
MI0000263	hsa-mir-7-1	9	-	85774483	85774592	No	*HNRNPK*	NM_002140	16	720
MI0000439	hsa-mir-23b	9	+	96887311	96887407	No	*C9orf3*	NM_032823	14	3961
MI0000440	hsa-mir-27b	9	+	96887548	96887644	No	*C9orf3*	NM_032823	14	3961
MI0000080	hsa-mir-24-1	9	+	96888124	96888191	No	*C9orf3*	NM_032823	14	3961
MI0006369	hsa-mir-1302-8	9	-	99165657	99165784	No	*KIAA1529*	NM_020893	9	1575
MI0000090	hsa-mir-32	9	-	110848330	110848399	No	*C9orf5*	NM_032012	14	12164
MI0003513	hsa-mir-455	9	+	116011535	116011630	No	*COL27A1*	NM_032888	10	3377
MI0003614	hsa-mir-601	9	-	125204625	125204703	No	*DENND1A*	NM_020946	20	19487
MI0000269	hsa-mir-181a-2	9	+	126494542	126494651	No	*NR6A1*	NM_001489	8	178748
MI0000683	hsa-mir-181b-2	9	+	126495810	126495898	No	*NR6A1*	NM_001489	8	178748
MI0000282	hsa-mir-199b	9	-	130046821	130046930	No	*DNM1*	NM_001005336	8	4047
MI0000471	hsa-mir-126	9	+	138684875	138684959	No	*EGFL7*	NM_201446	7	618
MI0003127	hsa-mir-511-1	10	+	17927113	17927199	No	*MRC1*	NM_002438	5	4482
MI0003128	hsa-mir-511-2	10	+	18174042	18174128	No	*MRC1*	NM_002438	5	4481
MI0008336	hsa-mir-1915	10	-	21825497	21825576	No	*C10orf114*	NM_001010911	1	803
MI0003616	hsa-mir-603	10	+	24604620	24604716	No	*KIAA1217*	NM_019590	2	160958
MI0003617	hsa-mir-604	10	-	29873939	29874032	No	*SVIL*	NM_021738	6	14526
MI0005760	hsa-mir-938	10	-	29931199	29931281	No	*SVIL*	NM_021738	1	65106
MI0003618	hsa-mir-605	10	+	52729339	52729421	No	*PRKG1*	NM_001098512	2	314391
MI0006374	hsa-mir-548f-1	10	-	56037640	56037723	No	*PCDH15*	NM_033056	2	136293

MI0003780	hsa-mir-1296	10	-	64802723	64802814	No	*JMJD1C*	NM_032776	2	115552
MI0006388	hsa-mir-1254	10	+	70189081	70189177	No	*CCAR1*	NM_018237	15	3628
MI0000826	hsa-mir-346	10	-	88014431	88014525	No	*GRID1*	NM_017551	2	157291
MI0000114	hsa-mir-107	10	-	91342484	91342564	No	*PANK1*	NM_148977	5	4418
MI0006349	hsa-mir-1287	10	-	100144965	100145054	Yes	*C10orf33*	NM_032709	8	N/A
MI0003621	hsa-mir-608	10	+	102724732	102724831	No	*SEMA4G*	NM_017893	3	4021
MI0006444	hsa-mir-1307	10	-	105144000	105144148	Yes	*USMG5*	NM_032747	2	N/A
MI0005758	hsa-mir-936	10	-	105797837	105797934	Yes	*COL17A1*	NM_000494	30	N/A
MI0003622	hsa-mir-609	10	-	105968537	105968631	No	*C10orf79*	NM_025145	3	11033
MI0006344	hsa-mir-548e	10	+	112738674	112738761	No	*SHOC2*	NM_007373	3	14648
MI0002467	hsa-mir-483	11	-	2111940	2112015	No	*IGF2*	NM_001007139	3	1700
MI0003623	hsa-mir-610	11	+	28034938	28035033	No	*KIF18A*	NM_031217	4	22260
MI0003624	hsa-mir-611	11	-	61316543	61316609	Yes	*C11orf10*	NM_014206	1	N/A
MI0008329	hsa-mir-1908	11	-	61339209	61339288	No	*FADS1*	NM_013402	1	3200
MI0006327	hsa-mir-1237	11	+	63892650	63892751	No	*RPS6KA4*	NM_001006944	11	101
MI0006354	hsa-mir-548k	11	+	69807709	69807824	No	*PPFIA1*	NM_003626	2	51964
MI0000261	hsa-mir-139	11	-	72003755	72003822	No	*PDE2A*	NM_002599	2	33501
MI0000808	hsa-mir-326	11	-	74723784	74723878	No	*ARRB1*	NM_004041	1	61547
MI0005543	hsa-mir-708	11	-	78790714	78790801	No	*ODZ4*	NM_001098816	1	142963
MI0006361	hsa-mir-548l	11	-	93839309	93839394	No	*MRE11A*	NM_005591	10	3572
MI0006379	hsa-mir-1244	12	+	12156153	12156237	No	*BCL2L14*	NM_138724	3	24444
MI0003626	hsa-mir-613	12	+	12808850	12808944	No	*APOLD1*	NM_001130415	1	60811
MI0005712	hsa-mir-920	12	+	24256622	24256696	No	*SOX5*	NM_152989	14	381190
MI0006353	hsa-mir-1291	12	-	47334494	47334580	No	*C12orf41*	NM_017822	9	764
MI0006355	hsa-mir-1293	12	-	48914192	48914262	No	*LIMA1*	NM_001113546	2	16921
MI0003628	hsa-mir-615	12	+	52714001	52714096	No	*HOXC4*	NM_014620	1	35744
MI0000811	hsa-mir-148b	12	+	53017267	53017365	No	*COPZ1*	NM_016057	1	15364
MI0006318	hsa-mir-1228	12	+	55874554	55874626	No	*LRP1*	NM_002332	49	72
MI0003629	hsa-mir-616	12	-	56199213	56199309	No	*DDIT3*	NM_004083	1	2663
MI0000750	hsa-mir-26a-2	12	-	56504659	56504742	No	*CTDSP2*	NM_005730	5	2025
MI0003630	hsa-mir-548c	12	+	63302556	63302652	No	*RASSF3*	NM_178169	1	74042
MI0006426	hsa-mir-1279	12	-	67953204	67953265	Yes	*CPSF6*	NM_007007	1	N/A
MI0006434	hsa-mir-1252	12	+	78337168	78337232	No	*SYT1*	NM_005639	6	90452
MI0003631	hsa-mir-617	12	-	79750443	79750539	No	*LIN7A*	NM_004664	4	34045
MI0003632	hsa-mir-618	12	-	79853646	79853743	No	*LIN7A*	NM_004664	1	48270
MI0003633	hsa-mir-619	12	-	107754813	107754911	No	*SSH1*	NM_018984	2	29275
MI0003634	hsa-mir-620	12	-	115070748	115070842	No	*MED13L*	NM_015335	2	125954
MI0006271	hsa-mir-1178	12	-	118635822	118635912	Yes	*CIT*	NM_007174	33	N/A
MI0003637	hsa-mir-623	13	+	98806386	98806483	No	*UBAC2*	NM_177967	5	27220
MI0006404	hsa-mir-1267	13	-	106981520	106981597	No	*FAM155A*	NM_001080396	1	654925
MI0006333	hsa-mir-1201	14	-	19864446	19864530	No	*CCNB1IP1*	NM_021178	3	1061
MI0000251	hsa-mir-208a	14	-	22927645	22927715	No	*MYH6*	NM_002471	29	519
MI0005570	hsa-mir-208b	14	-	22957036	22957112	No	*MYH7*	NM_000257	30	522
MI0003638	hsa-mir-624	14	-	30553603	30553699	No	*STRN3*	NM_001083893	1	69660
MI0006411	hsa-mir-548h-1	14	-	63631495	63631596	No	*SYNE2*	NM_182914	55	4467
MI0003639	hsa-mir-625	14	+	65007573	65007657	No	*FUT8*	NM_178155	2	105617
MI0006394	hsa-mir-1260	14	+	76802314	76802386	Yes	*NGB*	NM_021257	1	N/A
MI0000805	hsa-mir-342	14	+	99645745	99645843	No	*EVL*	NM_016337	3	25879
MI0001721	hsa-mir-431	14	+	100417097	100417210	Yes	*RTL1*	NM_001134888	1	N/A
MI0001723	hsa-mir-433	14	+	100417976	100418068	Yes	*RTL1*	NM_001134888	1	N/A
MI0000472	hsa-mir-127	14	+	100419069	100419165	Yes	*RTL1*	NM_001134888	1	N/A
MI0003133	hsa-mir-432	14	+	100420573	100420666	Yes	*RTL1*	NM_001134888	1	N/A
MI0000475	hsa-mir-136	14	+	100420792	100420873	Yes	*RTL1*	NM_001134888	1	N/A
MI0000287	hsa-mir-211	15	-	29144527	29144636	No	*TRPM1*	NM_002420	6	2782
MI0006323	hsa-mir-1233	15	-	32461562	32461643	No	*GOLGA8A*	NM_181077	20	81
MI0006323	hsa-mir-1233	15	-	32607783	32607864	No	*GOLGA8B*	NM_001023567	21	81
MI0003640	hsa-mir-626	15	+	39771075	39771168	No	*MGA*	NM_001080541	2	26115
MI0003641	hsa-mir-627	15	-	40279060	40279156	No	*VPS39*	NM_015289	2	8334
MI0006429	hsa-mir-1282	15	-	41873149	41873249	Yes	*SERF2*	NM_001018108	1	N/A
MI0005544	hsa-mir-147b	15	+	43512540	43512619	Yes	*C15orf48*	NM_197955	5	N/A
MI0006403	hsa-mir-1266	15	-	50356606	50356689	No	*MYO5C*	NM_018728	4	3153
MI0003642	hsa-mir-628	15	-	53452430	53452524	No	*CCPG1*	NM_020739	5	4903
MI0000486	hsa-mir-190	15	+	60903209	60903293	No	*TLN2*	NM_015059	51	12892
MI0006408	hsa-mir-1272	15	-	62841639	62841767	No	*RBPMS2*	NM_194272	1	23593
MI0003643	hsa-mir-629	15	-	68158765	68158861	No	*TLE3*	NM_005078	4	18372
MI0003645	hsa-mir-631	15	-	73433005	73433079	No	*NEIL1*	NM_024608	5	1036
MI0003679	hsa-mir-549	15	-	78921374	78921469	No	*KIAA1199*	NM_018689	28	94248
MI0006416	hsa-mir-1276	15	-	84114731	84114813	No	*KLHL25*	NM_022480	1	24944
MI0007074	hsa-mir-1469	15	+	94677494	94677540	No	*NR2F2*	NM_021005	2	1527
MI0003670	hsa-mir-662	16	+	760184	760278	Yes	*MSLNL*	NM_001025190	2	N/A
MI0006311	hsa-mir-1225	16	-	2080197	2080286	No	*PKD1*	NM_001009944	45	89
MI0000804	hsa-mir-328	16	-	65793725	65793799	No	*ELMO3*	NM_024712	7	111
MI0007259	hsa-mir-1538	16	-	68157212	68157272	Yes	*NFAT5*	NM_138714	16	N/A
MI0000456	hsa-mir-140	16	+	68524485	68524584	No	*WWP2*	NM_007014	17	2078
MI0008331	hsa-mir-1910	16	-	84332728	84332807	No	*C16orf74*	NM_206967	1	15689
MI0000078	hsa-mir-22	17	-	1563947	1564031	Yes	*C17orf91*	NM_032895	3	N/A
MI0000813	hsa-mir-324	17	-	7067340	7067422	No	*ACADVL*	NM_000018	9	405
MI0005559	hsa-mir-744	17	+	11925941	11926038	No	*MAP2K4*	NM_003010	3	14043
MI0006413	hsa-mir-548h-3	17	-	13387571	13387688	No	*HS3ST3A1*	NM_006042	1	103711
MI0006432	hsa-mir-1288	17	+	16126053	16126127	No	*PIGL*	NM_004278	2	65816
MI0003646	hsa-mir-33b	17	-	17657875	17657970	No	*SREBF1*	NM_001005291	17	474
MI0006273	hsa-mir-1180	17	-	19188412	19188480	No	*B9D1*	NM_015681	5	3410
MI0001445	hsa-mir-423	17	+	25468223	25468316	No	*CCDC55*	NM_032141	1	1215
MI0005715	hsa-mir-923	17	-	30502292	30502346	No	*UNC45B*	NM_001033576	16	2668

MI0000462	hsa-mir-152	17	-	43469526	43469612	No	*COPZ2*	NM_016429	1	740
MI0006335	hsa-mir-1203	17	-	43588788	43588872	No	*SKAP1*	NM_001075099	11	25130
MI0003820	hsa-mir-454	17	-	54569901	54570015	No	*FAM33A*	NM_182620	1	23762
MI0000745	hsa-mir-301a	17	-	54583279	54583364	No	*FAM33A*	NM_182620	1	23762
MI0003649	hsa-mir-634	17	+	62213652	62213748	No	*PRKCA*	NM_002737	15	1863
MI0003671	hsa-mir-548d-2	17	-	62898067	62898163	No	*PITPNC1*	NM_181671	9	154598
MI0003650	hsa-mir-635	17	-	63932187	63932284	No	*WIPI1*	NM_017983	12	4253
MI0003651	hsa-mir-636	17	-	72244127	72244225	Yes	*SFRS2*	NM_003016	2	N/A
MI0003681	hsa-mir-657	17	-	76713671	76713768	No	*AATK*	NM_001080395	7	1411
MI0000814	hsa-mir-338	17	-	76714278	76714344	No	*AATK*	NM_001080395	7	1411
MI0006385	hsa-mir-1250	17	-	76721591	76721703	No	*AATK*	NM_001080395	2	3161
MI0003778	hsa-mir-320c-1	18	+	17517469	17517556	No	*ABHD3*	NM_138340	5	19688
MI0000450	hsa-mir-133a-1	18	-	17659657	17659744	No	*MIB1*	NM_020774	9	18717
MI0000437	hsa-mir-1-2	18	-	17662963	17663047	No	*MIB1*	NM_020774	9	18717
MI0008191	hsa-mir-320c-2	18	+	20155648	20155697	No	*OSBPL1A*	NM_080597	21	14131
MI0008330	hsa-mir-1909	19	-	1767158	1767237	Yes	*REXO1*	NM_020695	15	N/A
MI0006316	hsa-mir-1227	19	-	2185061	2185148	No	*PLEKHJ1*	NM_018049	4	87
MI0003652	hsa-mir-637	19	-	3912412	3912510	No	*DAPK3*	NM_001348	5	2480
MI0005529	hsa-mir-220b	19	+	6446959	6447045	Yes	*TUBB4*	NM_006087	1	N/A
MI0006274	hsa-mir-1181	19	-	10375134	10375214	Yes	*CDC37*	NM_007065	1	N/A
MI0006328	hsa-mir-1238	19	+	10523798	10523880	No	*ATG4D*	NM_032885	8	82
MI0003653	hsa-mir-638	19	+	10690080	10690179	No	*DNM2*	NM_001005361	1	41333
MI0000242	hsa-mir-199a-1	19	-	10789102	10789172	No	*DNM2*	NM_001005361	6	7601
MI0007075	hsa-mir-1470	19	+	15421359	15421419	No	*WIZ*	NM_021241	7	1429
MI0003655	hsa-mir-640	19	+	19406872	19406967	No	*GATAD2A*	NM_017660	1	79200
MI0006407	hsa-mir-1270	19	-	20371080	20371162	No	*ZNF826*	NM_001039884	2	12742
MI0003656	hsa-mir-641	19	-	45480290	45480388	No	*AKT2*	NM_001626	1	19828
MI0000803	hsa-mir-330	19	-	50834092	50834185	No	*EML2*	NM_012155	1	482
MI0003657	hsa-mir-642	19	+	50870026	50870122	No	*GIPR*	NM_000164	7	2121
MI0005536	hsa-mir-220c	19	-	53755341	53755423	No	*SULT2B1*	NM_177973	6	23616
MI0003658	hsa-mir-643	19	+	57476862	57476958	No	*ZNF766*	NM_001010851	1	12478
MI0005757	hsa-mir-935	19	+	59177373	59177463	Yes	*CACNG8*	NM_031895	4	N/A
MI0006433	hsa-mir-1292	20	+	2581423	2581488	Yes	*NOL5A*	NM_006392	2	N/A
MI0000108	hsa-mir-103-2	20	+	3846141	3846218	No	*PANK2*	NM_024960	5	1619
MI0007262	hsa-mir-103-2-as	20	-	3846149	3846210	No	*PANK2*	NM_024960	2	1619
MI0008193	hsa-mir-1825	20	+	30289259	30289311	Yes	*POFUT1*	NM_015352	7	N/A
MI0003659	hsa-mir-644	20	+	32517791	32517884	No	*ITCH*	NM_031483	14	7825
MI0003183	hsa-mir-499	20	+	33041840	33041961	No	*MYH7B*	NM_020884	20	426
MI0006366	hsa-mir-1302-5	20	-	48664580	48664729	No	*FAM65C*	NM_080829	4	4796
MI0000651	hsa-mir-1-1	20	+	60561958	60562028	No	*C20orf166*	NM_178463	2	11322
MI0000451	hsa-mir-133a-2	20	+	60572564	60572665	Yes	*C20orf166*	NM_178463	3	N/A
MI0005763	hsa-mir-941-1	20	+	62021238	62021326	No	*DNAJC5*	NM_025219	1	33010
MI0005764	hsa-mir-941-2	20	+	62021545	62021633	No	*DNAJC5*	NM_025219	1	33010
MI0005765	hsa-mir-941-3	20	+	62021657	62021745	No	*DNAJC5*	NM_025219	1	33010
MI0008335	hsa-mir-1914	20	-	62043262	62043341	No	*UCKL1*	NM_017859	8	2443
MI0003662	hsa-mir-647	20	-	62044428	62044523	No	*UCKL1*	NM_017859	8	2443
MI0000101	hsa-mir-99a	21	+	16833280	16833360	No	*C21orf34*	NM_001005732	6	56899
MI0000064	hsa-let-7c	21	+	16834019	16834102	No	*C21orf34*	NM_001005732	6	56899
MI0000470	hsa-mir-125b-2	21	+	16884428	16884516	No	*C21orf34*	NM_001005732	6	56899
MI0003663	hsa-mir-648	22	-	16843634	16843727	No	*MICAL3*	NM_001122731	1	117393
MI0000482	hsa-mir-185	22	+	18400662	18400743	No	*C22orf25*	NM_152906	1	15512
MI0006443	hsa-mir-1306	22	+	18453581	18453665	Yes	*DGCR8*	NM_022720	2	N/A
MI0006348	hsa-mir-1286	22	-	18616657	18616734	No	*RTN4R*	NM_023004	1	24959
MI0006345	hsa-mir-548j	22	-	25281178	25281289	No	*TPST2*	NM_003595	1	45374
MI0000091	hsa-mir-33a	22	+	40626894	40626962	No	*SREBF2*	NM_004599	16	2470
MI0006384	hsa-mir-1249	22	-	43975499	43975564	No	*C22orf9*	NM_001009880	7	2953
MI0006441	hsa-mir-1308	X	-	21990180	21990233	No	*PHEX*	NM_000444	19	29175
MI0006378	hsa-mir-548f-5	X	-	32569512	32569597	No	*DMD*	NM_000109	11	29677
MI0003205	hsa-mir-532	X	+	49654494	49654584	No	*CLCN5*	NM_001127899	3	116999
MI0000484	hsa-mir-188	X	+	49654849	49654934	No	*CLCN5*	NM_001127899	3	116999
MI0003184	hsa-mir-500	X	+	49659779	49659862	No	*CLCN5*	NM_001127899	3	116999
MI0000762	hsa-mir-362	X	+	49660312	49660376	No	*CLCN5*	NM_001127899	3	116999
MI0003185	hsa-mir-501	X	+	49661070	49661153	No	*CLCN5*	NM_001127899	3	116999
MI0003684	hsa-mir-660	X	+	49664589	49664685	No	*CLCN5*	NM_001127899	3	116999
MI0003186	hsa-mir-502	X	+	49665946	49666031	No	*CLCN5*	NM_001127899	3	116999
MI0000100	hsa-mir-98	X	-	53599909	53600027	No	*HUWE1*	NM_031407	60	2460
MI0000068	hsa-let-7f-2	X	-	53600878	53600960	No	*HUWE1*	NM_031407	60	2460
MI0000760	hsa-mir-361	X	-	85045297	85045368	No	*CHM*	NM_000390	9	10071
MI0003667	hsa-mir-652	X	+	109185213	109185310	No	*TMEM164*	NM_032227	2	63181
MI0008333	hsa-mir-1912	X	+	113792275	113792354	No	*HTR2C*	NM_000868	2	112926
MI0003758	hsa-mir-1264	X	+	113793386	113793454	No	*HTR2C*	NM_000868	2	112926
MI0003938	hsa-mir-1298	X	+	113855906	113856017	No	*HTR2C*	NM_000868	2	112926
MI0008332	hsa-mir-1911	X	+	113904000	113904079	No	*HTR2C*	NM_000868	4	116548
MI0001637	hsa-mir-448	X	+	113964273	113964383	No	*HTR2C*	NM_000868	4	116548
MI0006419	hsa-mir-1277	X	+	117404385	117404462	No	*WDR44*	NM_019045	2	8886
MI0003836	hsa-mir-766	X	-	118664729	118664839	No	*6-Sep*	NM_145800	5	9147
MI0005756	hsa-mir-934	X	+	135460703	135460785	No	*VGLL1*	NM_016267	4	5628
MI0003189	hsa-mir-504	X	-	137577538	137577620	No	*FGF13*	NM_033642	3	67328
MI0000301	hsa-mir-224	X	-	150877706	150877786	No	*GABRE*	NM_021987	8	3977
MI0001733	hsa-mir-452	X	-	150878756	150878840	No	*GABRE*	NM_021987	8	3977
MI0000111	hsa-mir-105-1	X	-	151311347	151311427	No	*GABRA3*	NM_000808	1	86548
MI0003763	hsa-mir-767	X	-	151312549	151312657	No	*GABRA3*	NM_000808	1	86548
MI0000112	hsa-mir-105-2	X	-	151313540	151313620	No	*GABRA3*	NM_000808	1	86548
MI0006277	hsa-mir-1184	X	-	153768829	153768927	No	*F8*	NM_000132	22	32848

MI0006277	hsa-mir-1184	X	-	154265943	154266041	Yes	*F8A1*	NM_012151	1	N/A
MI0006277	hsa-mir-1184	X	+	154340372	154340470	Yes	*F8A1*	NM_012151	1	N/A
