# Supplementary material for: A potential role for intragenic miRNAs on their hosts' interactome
Source: BMC Genomics. 2010 Oct 1;11:533. doi: 10.1186/1471-2164-11-533 (PMC3091682; doi:10.1186/1471-2164-11-533)
Supplement: Additional file 2 — Distribution of intragenic miRNAs. Additional file 2 contains an additional barplot showing the distribution of intronic miRNAs across their hosts' introns, as well as a theoretically expected distribution taking intron frequency and size into consideration. The first figure on page 1 shows a barplot of expected and observed distribution of intragenic miRNAs across their hosts' interactome. The second figure is a repetition of Figure 1b, for better comparison. The second page contains the underlying data in table format. [file 1471-2164-11-533-S2.PDF]

Distribution of miRNAs across Host Gene Introns

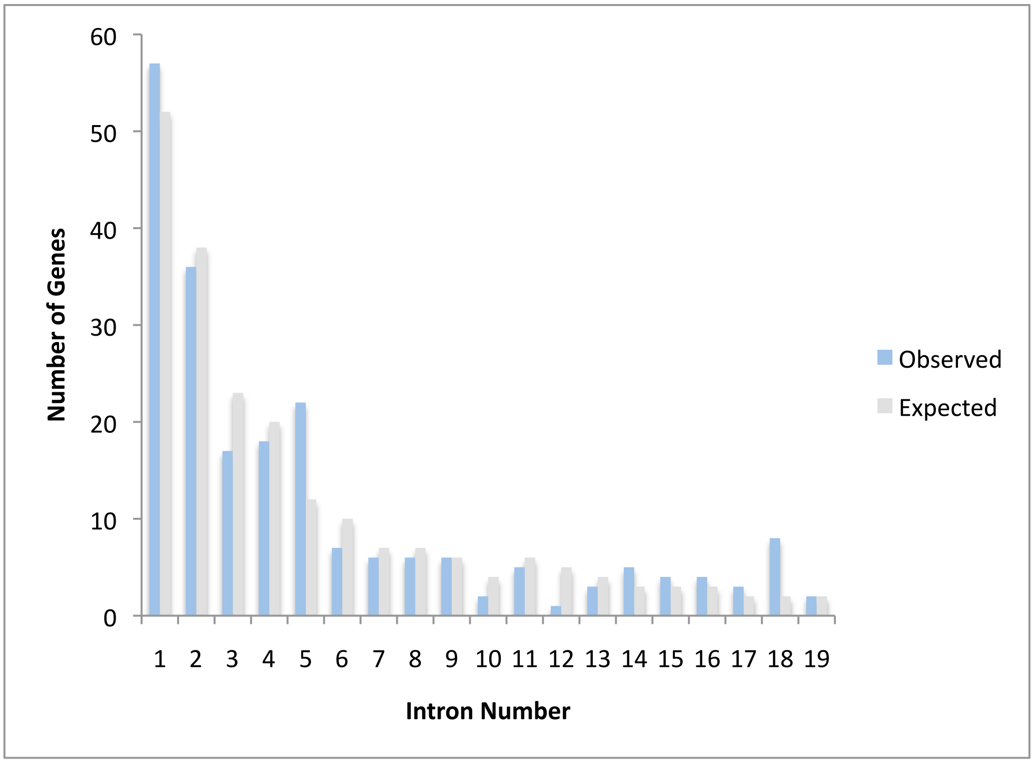

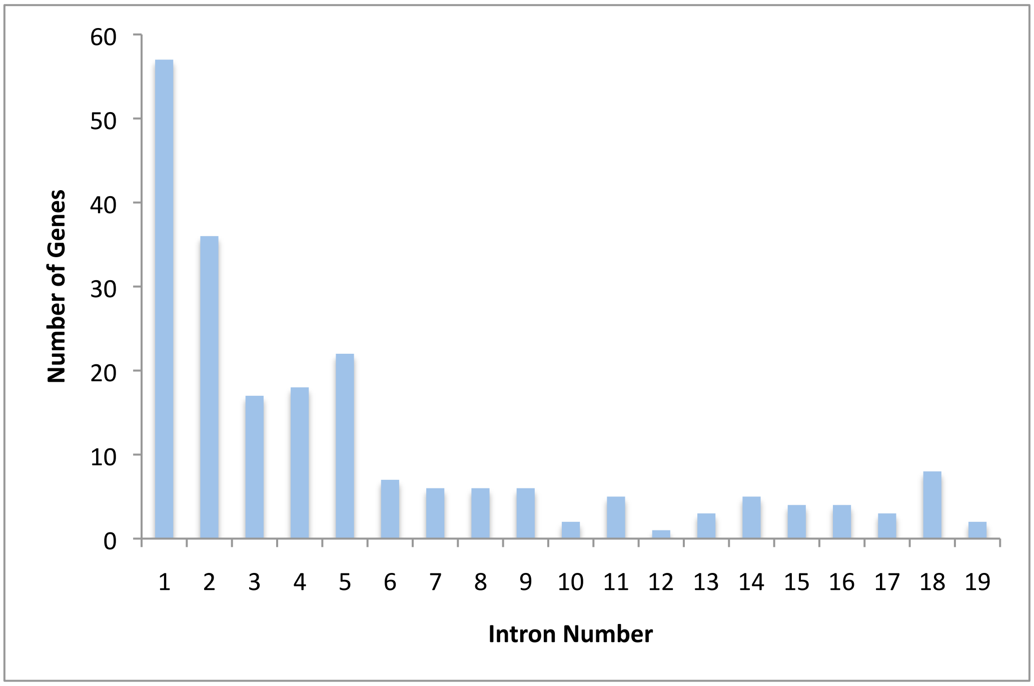

Intron	Probability (normalized by Intron Count and Length)	Observed	Expected
1	0.22763832	57	52
2	0.16584427	36	38
3	0.09914632	17	23
4	0.08809541	18	20
5	0.05424016	22	12
6	0.04270313	7	10
7	0.02951425	6	7
8	0.02865113	6	7
9	0.02420967	6	6
10	0.01747431	2	4
11	0.02425617	5	6
12	0.02329309	1	5
13	0.01667473	3	4
14	0.01250191	5	3
15	0.01167813	4	3
16	0.01357904	4	3
17	0.00859208	3	2
18	0.00794428	8	2
19	0.00816208	2	2
20	0.0097078	3	2
21	0.00522884	3	1
22	0.00569624	2	1
23	0.00442481	0	1
24	0.00244285	0	1
25	0.00462643	0	1
26	0.00403074	0	1
27	0.00259172	0	1
28	0.00230005	0	1
29	0.00312252	1	1
30	0.00197901	1	0
31	0.0011064	0	0
32	0.00130089	0	0
33	0.00114629	0	0
34	0.00142463	0	0
35	0.00055822	0	0
36	0.00073724	0	0
37	0.00174385	0	0
38	0.00079874	0	0
39	0.00102446	0	0
40	0.0005816	1	0
41	0.00121192	0	0
42	0.0012971	1	0
43	0.00218786	0	1
44	0.0065795	0	2

45	0.00153313	1	0
46	0.00048602	0	0
47	0.00177196	0	0
48	0.00138025	0	0
49	0.00098197	1	0
50	0.00157978	0	0
51	0.00148958	1	0
52	0.00136185	0	0
53	0.00060676	0	0
54	0.00086993	0	0
55	0.00312269	0	1
56	0.00034518	0	0
57	0.00063737	0	0
58	6.5879E-05	0	0
59	0.00099161	0	0
60	0.00249459	1	1
61	0.00095406	1	0
62	0.00160555	0	0
63	0.00100358	0	0
64	0.00036727	0	0
65	0.00071252	0	0
66	0.00054904	0	0
67	0.00057113	0	0
68	0.0001561	0	0
69	8.7101E-05	0	0
70	0.00014659	0	0
71	0.00022272	0	0
72	0.00014447	0	0
73	0.00025294	0	0
74	0.0006055	0	0
75	5.4789E-05	0	0
76	0.00033859	0	0
77	0.00021047	0	0
78	0.00014413	0	0
79	1.5173E-05	0	0
80	1.9747E-05	0	0
81	2.0189E-05	0	0
82	1.3599E-05	0	0
83	2.1861E-05	0	0
84	3.3198E-06	0	0
85	3.9346E-06	0	0
86	2.7296E-06	0	0
87	2.4099E-06	0	0
88	3.7378E-06	0	0
