# Supplementary figures and images for: A potential role for intragenic miRNAs on their hosts' interactome
Source: BMC Genomics. 2010 Oct 1;11:533. doi: 10.1186/1471-2164-11-533 (PMC3091682; doi:10.1186/1471-2164-11-533)

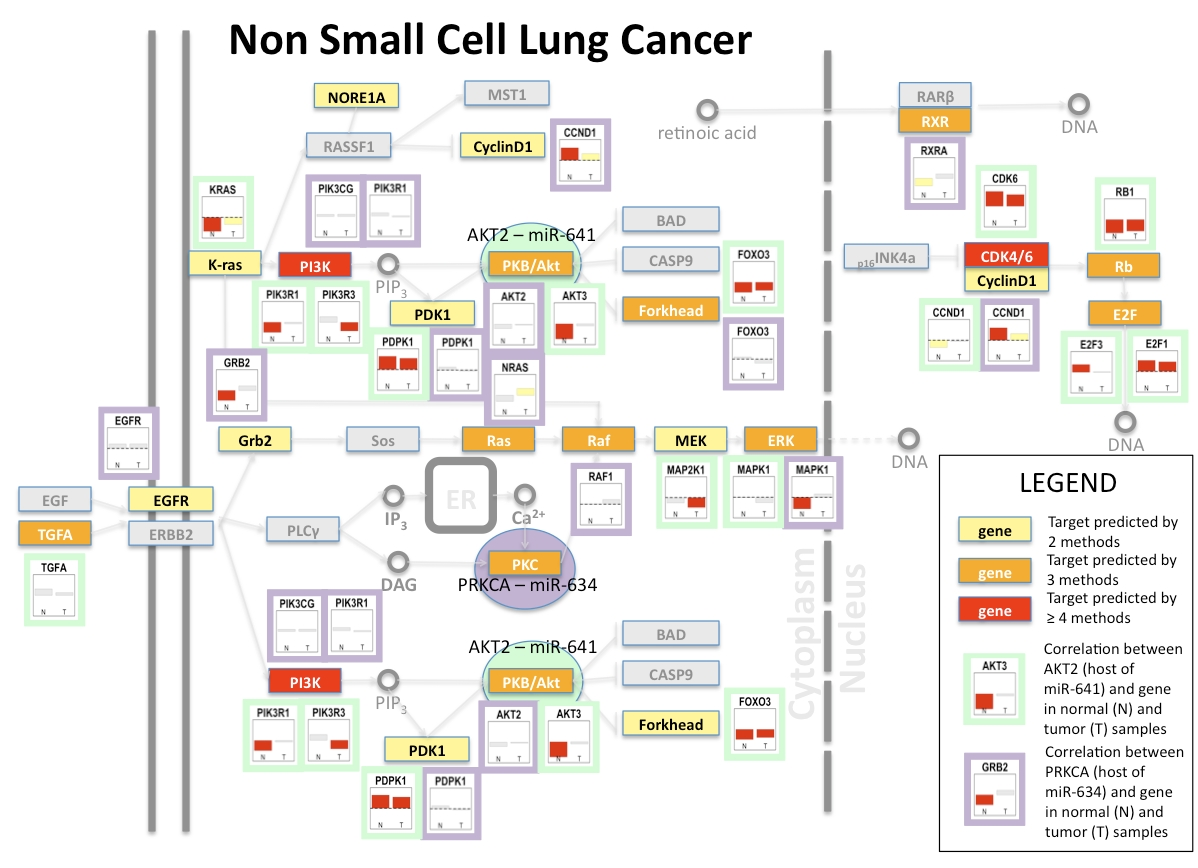

Supplement: Additional file 4 — Non Small Cell Lung Cancer. The figure is analogous to Figure 2, for a non small cell lung cancer mRNA expression microarray dataset. [file 1471-2164-11-533-S4.TIFF]
